# Quantum-Based Creative Generation Method for a Dancing Robot

**DOI:** 10.3389/fnbot.2020.559366

**Published:** 2020-12-01

**Authors:** Peng Mei, GangYi Ding, QianKun Jin, FuQuan Zhang, YangFan Jiao

**Affiliations:** ^1^Digital Performance and Simulation Technology, School of Computer Science & Technology, Beijing Institute of Technology, Beijing, China; ^2^Fujian Provincial Key Laboratory of Information Processing and Intelligent Control, Minjiang University, Fuzhou, China; ^3^Beijing Wanshide Technology Co., Ltd., Beijing, China

**Keywords:** creative generation, quantum simulation, information fidelity, M-3DQKG, QGAN, robot trajectory

## Abstract

In this paper, we propose a creative generation process model based on the quantum modeling simulation method. This model is mainly aimed at generating the running trajectory of a dancing robot and the execution plan of the dancing action. First, we used digital twin technology to establish data mapping between the robot and the computer simulation environment to realize intelligent controllability of the robot's trajectory and the dance movements described in this paper. Second, we conducted many experiments and carried out a lot of research into information retrieval, information fidelity, and result evaluation. We constructed a multilevel three-dimensional spatial quantum knowledge map (M-3DQKG) based on the coherence and entangled states of quantum modeling and simulation. Combined with dance videos, we used regions with convolutional neural networks (R-CNNs) to extract character bones and movement features to form a movement library. We used M-3DQKG to quickly retrieve information from the knowledge base, action library, and database, and then the system generated action models through a holistically nested edge detection (HED) network. The system then rendered scenes that matched the actions through generative adversarial networks (GANs). Finally, the scene and dance movements were integrated, and the creative generation process was completed. This paper also proposes the creativity generation coefficient as a means of evaluating the results of the creative process, combined with artificial brain electroenchalographic data to assist in evaluating the degree of agreement between creativity and needs. This paper aims to realize the automation and intelligence of the creative generation process and improve the creative generation effect and usability of dance movements. Experiments show that this paper has significantly improved the efficiency of knowledge retrieval and the accuracy of knowledge acquisition, and can generate unique and practical dance moves. The robot's trajectory is novel and changeable, and can meet the needs of dance performances in different scenes. The creative generation process of dancing robots combined with deep learning and quantum technology is a required field for future development, and could provide a considerable boost to the progress of human society.

## Introduction

Robot trajectory calculations have always been an essential subject of scientific research. Nevertheless, directly programming a robot takes much debugging time and development costs. In this paper, computer simulation technology and a quantum modeling method are used to generate dance movement creativity that meets the performance and calculation of the robot trajectory data. The data are simulated and rehearsed by a digital twin to realize the robot's dance performance. Simulation technology has been widely used since the 20th century (Zeigler et al., 2000). With the maturity of computer technology, simulation technology has been further popularized through simulation software. Now, simulation technology has been widely used in stage performance (Bilbao, 2009; Niedenthal et al., 2010; Jaber et al., 2019), event scheduling (Colella, 2011; Arima, 2015), emergency response (Merién et al., 2010), military training (Liang et al., 2001; Machado et al., 2015; Ma et al., 2016), aerospace (Zipfel and Schiehlen, 2001; Jha et al., 2015), industrial manufacturing (Buyya and Murshed, 2010; Yamaguchi et al., 2016; Santipanusopon and Worawattanaparinya, 2019; Taheripour et al., 2019), technology research and development (Boyd and Bruns, 2001; Rapaport et al., 2002; IEEE, 2010; Binder and Heermann, 2014; Moin, 2018; Yingying et al., 2018), and many other fields. The simulation process has many commonalities, and these commonalities can be found in the application of simulation technology in various fields (Bucklew, 2010). As one of the tools to assist us in completing the planning, it has an essential and irreplaceable role in insignificant events. For example, the US military simulation system (Zhou et al., 2015) to the global strategic military defense system evaluation (Burger and Jenkins, 2013). Simulation is used to construct mathematical and physical models through computer modeling tools (Kasztenny and Kezunovic, 2002). Its purpose is to help people discover the problems that may be encountered during the development of an event (Biolek et al., 2019). Therefore, this paper attempts to use simulation technology to solve the problem of dance creativity for a dancing robot in order to assist people in the creative generation process to make decisions that meet their needs. Simulation technology can realize data interconnections between the real world and the virtual world by establishing a digital twin relationship with the robot. This paper uses simulation technology to generate dance robot data, including body movement data on points and spatial trajectory movement data. This paper establishes the usefulness of the multilevel three-dimensional spatial quantum knowledge map (M-3DQKG) knowledge retrieval model by determining the domain of the requirement ontology: to retrieve creative points according to creative needs to generate a creative pool. Using the creative pool as the original data, M-3DQKG is updated through quantum generative adversarial networks (QGANs) learning. The retrieval weight of this model is allocated according to the probability branch model. The knowledge retrieval process is performed according to weight. The generated ideas are evaluated by means of the subjects' electroencephalographic (EEG) data and creative generation coefficient (CGC) calculation results. The whole process described in this paper simulates the creative generation process, which provides a quick and effective method of enhancing the richness of creativity. Migrating the simulation data to the dancing robot can operate the robot's dance performance and trajectory movement.

Section Related Work is an introduction to related work. Section Method mainly introduces the critical technologies of dance creative movement generation, including the M-3DQKG model, probability branch model, creative generation model, information fidelity calculation method, and system creative generation ability evaluation method. Section Results presents an analysis and discussion of the experimental results. The last section is the conclusion and future prospects.

## Related Work

This section will introduce related work and the key technologies involved in this field. As a relatively new research direction, creativity generation is not widely understood by many people. This section first introduces the concepts of originality, creativity, and creativity generation, as well as the cognition of these concepts by related researchers. Next, this section will introduce the essential means of realizing creative generation–computer simulation technology. This technology can complete the creative process in a virtual environment and transfer the simulated data to the dancing robot to complete dance performances and trajectory movements. In the simulation process, the three links of knowledge map construction, machine learning network, and result evaluation are all integrated into quantum modeling methods. This accelerates the creative generation process from a new perspective and enriches the creative results.

### Originality, Creativity, and Creative Generation

Originality is new original ideas. Creativity is the ability to generate originality. And creative generation is a flow of operations that span time to create originality.

Originality is a new abstract thinking or action based on people's cognition of things. It cannot get rid of cognition and exists alone, and human perceptions are very different. Therefore, there is no unified rule for the definition of creativity or for the evaluation criteria of creativity. At this stage, although the creative process is complicated, and the tasks are numerous, people's demand for it is becoming greater. Not only that, but with the development of society in the future, this demand will continue to increase. This paper starts with meeting people's creative needs, breaking the single boundary of people's cognition, and proposes a method that can be quantitatively evaluated.

Originality arises as a new thing, but it is in fact a recombination of old elements. People's insight into the relationship between elements is the basis for generating new combinations. The creative generation process of the brain is the perfect cooperation between knowledge and neurons. Simulating the creative generation process of the human brain requires two conditions. Pezzulo et al. (2013) proposed that one is widely generated and frequently changed, and the other is summarized and filtered from it. The focus of the former is to continuously acquire knowledge, analyze knowledge, use trial and error, and correct. The focus of the latter is to emphasize the method of selection. In 1960 Campbell proposed the theory that creativity requires blind change and selective retention, exploring the generation of creativity (Simonton, 2012). These ideas have become the key technical points for computers to complete the creative generation process.

The process of creative generation is differently affected by a person's personality (Shamay-Tsoory et al., 2011), intelligence level (Kim, 2016), educational situation (Calignano and Jsendal, 2018), and creative environment (Saorín et al., 2017). One study (Steele et al., 2018) proposed neural and cognitive models to balance the influence of cognition on creativity. This paper explores the automation and intelligence of the creative generation process so that the creativity of the system is maintained at a stable level, and the results are free from the influence of individual differences. Therefore, a dance movement creative generation model is proposed to complete the creative generation process without human intervention and environmental influence. Studies have found that the internal role of emotion is the basis for the association between people's creativity and intuitive thinking (Haijuan et al., 2018). Positive emotions can enhance people's original ideas (Rooij et al., 2017) and make people work hard on simple tasks that are fun and stupid. Negative emotions make people work harder on serious and essential complex tasks (Friedman et al., 2007). Therefore, people use a creative process with emotion as the dominant position (Agnoli and Corazza, 2019). This paper simulates the emergence of emotion-led creativity based on the random nature of quantum modeling. The purpose is to increase the novelty of originality. The improvement of this technology has become one of the motivations for computers to complete the design of creative generative models.

This paper takes creative needs as the starting point and realizes the unity of opposites between creative thinking and critical thinking (Diyanni, 2014; Rivas, 2017): jump out of the shackles of creativity and standardization (Oliver et al., 2019) to give freedom to people's creative ability (Robinson, 2011; Jonason et al., 2015). The computer has become a capable means of giving full freedom to creativity with its plasticity, stability, upgradeability, and supercomputing power. Knowledge retrieval and learning networks break the upper limit of human creativity so that creativity is not affected by individual education, intelligence, experience, environment, needs, and other factors. The movement data generated by dance creativity take the virtual human in the simulation system as the carrier and are digitally twinned to the physical robot to drive the robot's limb movement and trajectory calculations to enhance the expressive power of the dancing robot.

### Computer Simulation Technology

Computer simulation technology emerged in the 1950's and was transformed from analog to digital simulation in the 1960's (Gould and Tobochnik, 2007). The process of simulation transforms the uncertainty of an event into a representation of the overall level of the event through modeling (Roux et al., 1973). Its purpose is to clarify the development trend of things and eliminate the vague understanding of the connections between things (Mustafee et al., 2018). Simulation is used to simulate and map real events. The modeling method is the primary tool for computer simulation to enhance people's understanding of things (Rozenblit, 2015).

Based on the advantages of computer simulation, researchers have made many attempts in the field of creative generation. In their research, Li proposed combining original thinking models with intuitive thinking methods such as the Theory of Inventive Problem Solving (TRIZ) and brainstorming (Li et al., 2003), through computer-aided product innovation. Computer simulation technology uses a neural network to process voice (Hinton et al., 2012; Graves et al., 2013; Muckenhirn et al., 2017), image (Sjöström et al., 2015; Lin et al., 2016; Hu et al., 2018; Sana et al., 2018), and video (Matta, 2008; Han et al., 2017) data and extracts the characteristics of the simulated object. The simulation system performs three-dimensional modeling based on the characteristic data. The application of GANs in the field of computer simulation has also accelerated the process of data processing and learning. Although different GANs methods are based on various convolutional neural network (CNN) frameworks (Wang et al., 2019), experiments by Goodfellow et al. have proved the potential of this framework through qualitative and quantitative evaluation of the generated samples (Goodfellow et al., 2014). Similarly, the apparent advantage of computer simulation technology improves the credibility of evaluating simulation results. Zheng et al. (2017) made some attempts in their paper, assigning recognition results into five categories and generating results through six-category detection. Recently, the University of California, Berkeley, has made significant progress (Xu et al., 2020). They proved the advantages of GANs evaluation results in the simulation system. In addition, an essential method of computer simulation technology is the modeling method. Moore and Cain (William and Ralph, 2015) and others pointed out the vital role of mathematical ability and logical methods in the process of computer simulation creative generation. Zhang et al. (2018) proposed a computable model of creative generation, which provides a theoretical basis for the digitization of the creative generation process. The construction of the simulation environment of computational simulation technology provides a simulation platform for the performance of a dancing robot. This is a great help in saving development costs and shortening the time required for choreography.

Originality needs to be evaluated in a consensus model (Mumford and Mcintosh, 2017). The computer simulation system uses the measurement of brain waves (EEG) (Matthias et al., 2010) to obtain people's quantitative indicators of creative results. At the same time, the scope of innovation is defined in artificial intelligence (Gazoni, 2016). The process of creative generation is the divergence of thinking information, but it does not mean that its expression function does not converge. When the system complexity reaches a saturation point, from the perspective of thermodynamics, energy will be released at this time. At this time, the function tends to converge. This paper defines the release range of this idea in the simulation system as the “emergence value range” (EVR). It also proposes the CGC as a means of measuring the creativity of the system. The evaluation method is realized by the quantum modeling method.

This paper gives full play to the advantages of computer simulation technology in visualization, modeling, and evaluation. It generates robot dance creativity and trajectory calculation results that meet people's needs. It drives dance robot performance through digital twin technology. This process realizes the point data sharing of robot dance performance and trajectory movement. Computer simulation technology combined with quantum modeling methods quantitatively evaluate the robot dance creativity and trajectory calculation capabilities.

### Quantum Modeling Method

Since the introduction of quantum theory, it has been widely concerned in all walks of life. Nowadays, it is not only a modeling method and calculation theory in traditional physics but also an essential tool for problem-solving in fields such as mathematics (Jayantika et al., 2019), chemistry (Nalewajski and Roman, 2017), the life sciences (Han et al., 2001), and computer science (Bennett and DiVincenzo, 2000; Schramm, 2017; Aerts et al., 2019). For computer simulation, the superposition and entanglement of quantum theory mean that it plays a pivotal role. Quantum computing can be realized by using the properties of quantum superposition (Gyongyosi and Imre, 2019), and the applications of quantum entanglement are mainly in the fields of quantum teleportation, quantum encryption, and quantum dense coding. With the help of thermodynamic principles, the complexity of a quantum system can be calculated by entropy. The complexity theory of quantum computing is one of the fundamental theories of quantum computer science (Zhang et al., 2016a). This paper uses the quantum superposition property and GANs to construct QGANs to accelerate the data-processing and -learning process. This paper uses quantum theory, the superposition state, and entangled states in building the knowledge storage and retrieval structure of M-3DQKG. At the same time, by calculating the complexity of the quantum system, combined with CGC, it is possible to evaluate the creative generation ability of the simulation system.

If this paper transforms information into quantum state information, the information at this time is a superposition state. Then the form of the quantum state information is stored in each qubit. The superposition state makes the storage capacity of data much more important than traditional storage methods. This can prompt M-3DQKG to store more information in a shallower structure. Moreover, the qubit in the calculation is the superposition of 0 and 1, which makes quantum computation much faster than other classical algorithms (Knill et al., 2001). This can promote the QGAN based on this to have a better learning ability. More information stored and retrieved by the algorithm can make the trajectory calculation of the dancing robot faster, and the body language more abundant. We use creative analogy (Christensen and Ball, 2016) to combine newly acquired innovative thinking skills with normal scientific thinking processes and use proven techniques to expand the ability of humans to generate original ideas (Ness, 2012). Kitaev et al. (2011) and others introduced the new theory of quantum computing. The information of classical systems can be calculated with the help of quantum systems and their properties (Nielsen and Chuang, 2007; Roy et al., 2017), so as to realize the research of classical computing theory and quantum mapping. This also provides new ideas for improving the computing power of classical systems.

In the process of studying quantum entanglement, we were surprised to find that the coherence of the input state and the entanglement of the output state are quantitatively equivalent (Berta et al., 2015). The conversion between the coherent state and the entangled state means that the quantum coherent state can be measured by the entangled state (Plenio and Virmani, 2011). Nevertheless, we also found that quantum entanglement and quantum teleportation (Prakash, 2010) are effective means of quantum information transmission. Before the development and popularization of quantum computers, we could use computer simulation technology to simulate quantum models.

The entropy of the complexity of a quantum system is equivalent to the quantum information entropy (Edward, 2015). The linear characteristics of quantum systems make it possible to calculate the information entropy of quantum systems. As the quantum system complexity can be calculated through entropy (Deng et al., 2012), if the mapping relationship between the microscopic quantum system and the macroscopic classical system can be found (Neill et al., 2016), it may be possible to solve the problem that the complexity of the classical system cannot be evaluated owing to non-linear characteristics (Jami and Labbafi, 2017; Madhok et al., 2018).

According to the viewpoint of quantum information theory, quantum entanglement is a kind of physical existence with a measurement effect (Yu and Song, 2004; Li et al., 2010; Shota et al., 2017). Through the measurement and calculation of multiple dimensions of quantum theory (Heydari, 2006; Mintert and Buchleitner, 2007; Chernyavskiy, 2009), the purpose of system complexity evaluation can be achieved. Therefore, an entangled quantum system with strong operability, useful calculations, and containing mixed and pure states is valuable. This is conducive to quantitative evaluation to ensure the effectiveness of the dance robot's trajectory and the practicality of the creative action process.

### Summary

The main content of this paper is to use computer simulation technology to realize the generation and trajectory calculation of dance robot dance creativity. The system builds the M-3DQKG structure based on the quantum modeling simulation model to achieve information storage and retrieval. Under the premise of information fidelity, a variety of neural networks are used to accelerate the creative generation process and increase its richness. This paper uses the quantum system linear method and EEG test to quantify and evaluate the creative ability and creative results of the system. The simulation system uses digital twin technology to copy the simulation output results that meet the needs of the dancing robot. These data will drive the trajectory and dance movements of the dancing robot.

## Method

**Figure 1:**
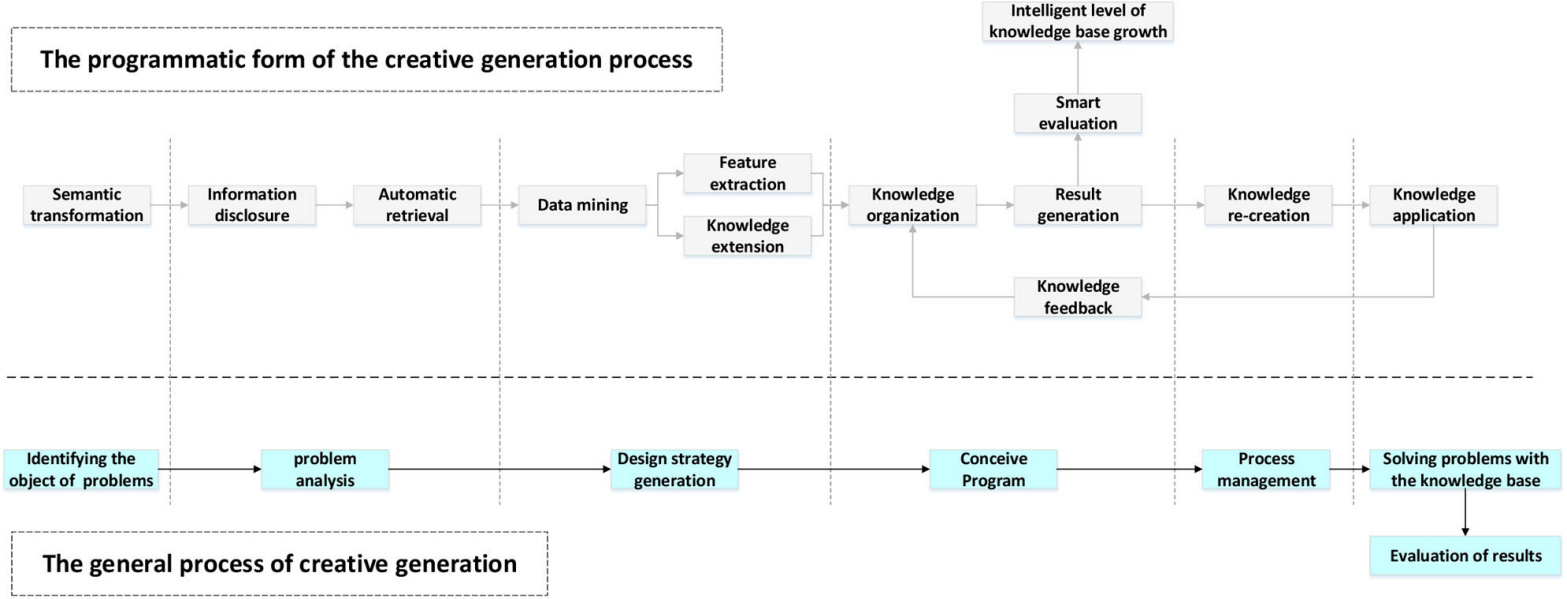
Comparison between the general process of creative generation and the programmatic form.

This paper proposes a modeling and simulation method to enhance the creative generation effect and uses this method to realize the action arrangement and trajectory calculation of the dancing robot in a particular scene. As shown in Figure 1, the general process of idea generation has many links and requires a lot of human resources and time to complete. This leads to very low efficiency in the proposal of creative generation schemes. Programming the creative generation process will be an essential means of improving efficiency and reducing costs.

**Figure 2:**
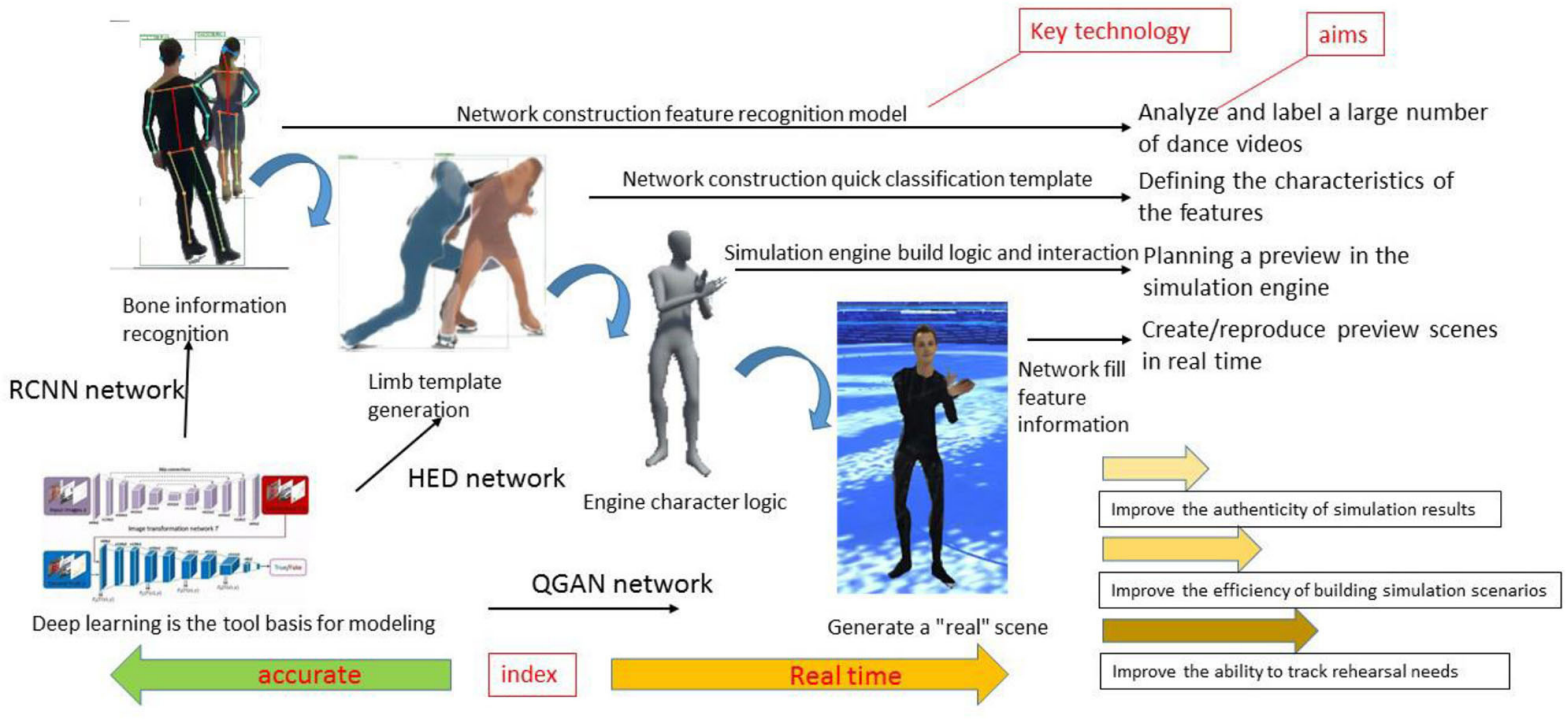
The main links and key techniques of creative dance movements. HED, holistically nested edge detection; R-CNN, regional convolutional neural network; QGAN, quantum generative adversarial network.

The generation of dance creativity mainly involves four main links: skeleton movement extraction, movement template generation, three-dimensional (3D) model production, and the integration of characters and scenes, as shown in Figure 2. The region with convolutional neural networks (R-CNNs) recognizes characters' actions and movement tracks in text, sound, and images, and extracts bone information and displacement point information. The holistically nested edge detection (HED) network generates abstract models of virtual human limbs as action templates by extracting information. In the 3D engine, we bind the generated action template and bone attributes to the character model and drive the character model to complete the dance motion simulation. The QGAN network generates performance scenes based on the information extracted by R-CNNs.

To enhance the practicality and novelty of the results of dance creativity, we have made new attempts to increase the capacity of information storage and the efficiency of knowledge retrieval. We start from the creative needs, extract the keywords of the needs, and form the ontology domain, abstract model, and the weight index of the demand knowledge. The ontology domain is used to define the object to be creative and the constraint range of the object's actions, scenes, and expressions. The abstract model is a data model generated by the ontology domain to describe the virtual person. The demand knowledge weight index is a weight table defined according to the frequency and importance of the keywords in demand. Moreover, we extract dance videos, images, and music information from R-CNNs to form an action library. The action library stores the body movement data and the running track. Based on the properties of the quantum superposition state and entangled state, we build M-3DQKG result storage and retrieval knowledge based on action feature tags.

To ensure the accuracy of the body template and the rationality of the fusion of characters and scenes, we have carried out some research into information fidelity and optimizing the creative generation process. We put forward the calculation method of information fidelity and related parameters for each link. Moreover, we optimize the closure of the information flow of the two processes of action generation and scene generation in the creative generation process. Our purpose in doing this is to keep information from being disturbed and lost by the external environment. We use the information degree to continuously measure the information changes of the system and provide an available reference for the final creative result.

To evaluate the degree of creativity of the creative generation results, we propose a CGC linear evaluation method based on measuring the information entropy of the quantum system. This combines EEG results to evaluate creative results. CGC is the mapping value of the quantum system information complexity in the classical system. We calculate the complexity of the quantum system based on the equivalence relationship between quantum entanglement entropy and information entropy. We use the principle of thermodynamics to calculate the relationship between the information entropy of the quantum system and the classical system and realize the mapping of linear quantum systems to non-linear classical systems. We defined the critical value of the convergence of the CGC description function as the EVR to evaluate the creative generation ability of the system. In addition, we invited volunteers to take an EEG test while watching creative generation videos. We marked creative points based on EEG peaks. Creative points are used as knowledge labels for neural network learning. The result of learning is an update of the structure and weight of M-3DQKG. Finally, the purpose is to achieve the convergence of the CGC description function.

This paper takes the generation of dance movements as an experimental case and visualizes the generated dance movement videos in a 3D simulation engine through character modeling and scene fusion. The system will meet the simulation results of the needs assessment to produce dance robot bones, joints, and displacement data. These data are transferred to the robot, and, finally, used to realize the dance performance and trajectory calculation of the dancing robot.

### Knowledge Retrieval Model

**Figure 3:**
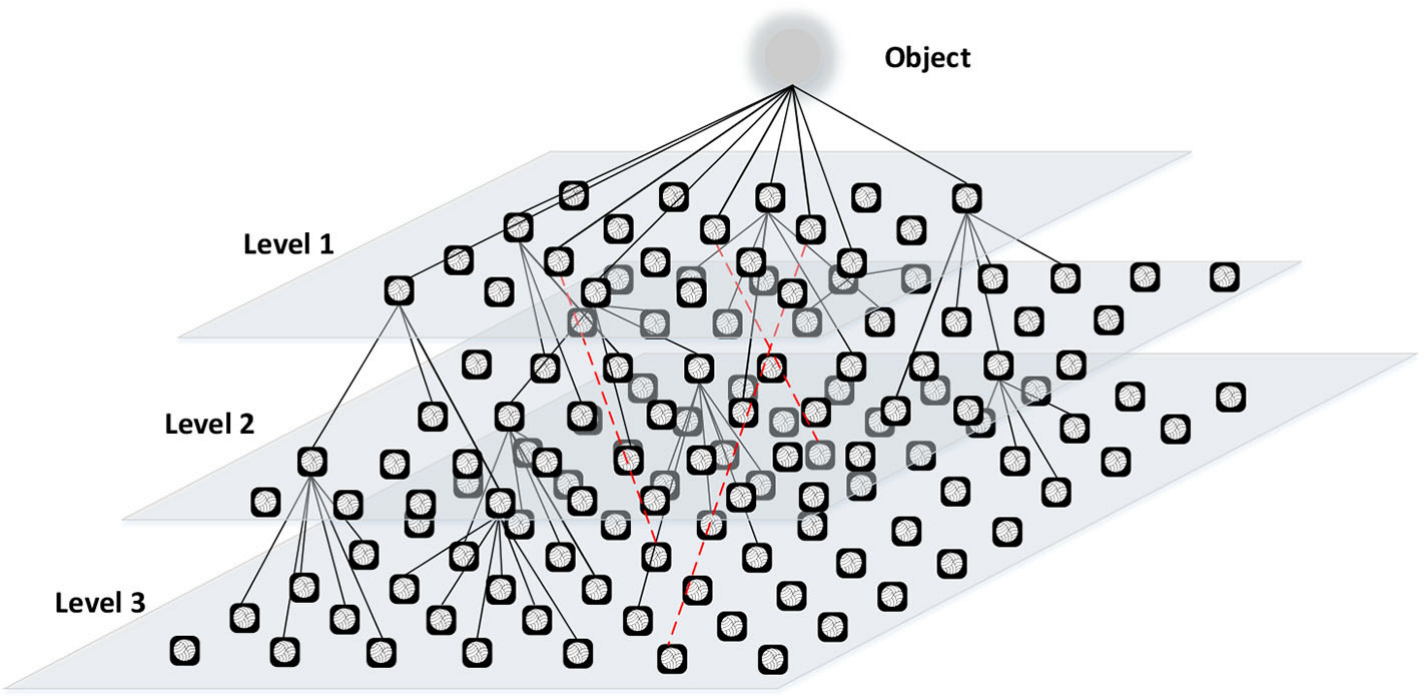
Examples of quantum knowledge graphs.

Knowledge retrieval of the creative generation is based on the knowledge graph. We propose the M-3DQKG model, which is used to construct the knowledge graph of the research object. Knowledge is a description of the attributes of the research object. Each node is defined as a qubit to store a unit of knowledge. The knowledge of a qubit is in a superposition state, which increases the data capacity of the knowledge graph. We divide knowledge into levels and determine the attribution or relationship of the upper and lower levels. Each layer forms an array of qubits. With the increase and connection of the qubits storing the knowledge unit points, the system creates an intricate knowledge network, as shown in Figure 3. When acquiring knowledge, knowledge units are retrieved one by one from the first level to deeper levels according to the connection relationship between qubits. Among them, the knowledge unit is locally entangled. A red dotted line connects the two entangled qubits in Figure 3. The generation of entanglement is determined based on the coherent state of the knowledge points stored in the qubit. When a knowledge unit in an entangled state is retrieved, we think that a measurement has been made. At this time, the state of the qubit is determined, and the superposition state of the qubit disappears. The information from the state change is instantly transmitted to the qubit at the other end through entanglement. The new search starts from this qubit, which realizes the cross-level search of the knowledge unit. The M-3DQKG model increases the diversity of knowledge unit retrieval. This model not only enriches the knowledge structure but also promotes faster information transmission.

In the process of retrieving knowledge based on the M-3DQKG model, the connection relationship between qubits is assigned weights according to the characteristics of the research object. Furthermore, we define the knowledge unit of the qubits in an entangled state. If one of the qubits is retrieved, the other qubit must be extracted. Therefore, the connection between them is assigned a weight of 1. The initial structure of the knowledge graph is based on research-on demand ontology (Jing et al., 2013; Zhang et al., 2016b). This ensures that the creative generation results remain valid. We extract the demand ontology and calculate the ontology domain of the idea generation so that the idea generation process always meets the constraints of the demand.

The learning network extracts keywords from the input demand information. The main research object is defined as the central ontology or primary ontology, and the secondary object is defined as the related ontology or secondary ontology. The ontology is the carrier of creative generation, and the keywords are bound to the ontology as attributes of the ontology. After that, the keywords are sorted according to their importance, and the sort number is marked. Take the expression rules in Table 1 as an example of designing a sorting number labeling algorithm.

**Table 1:** Ontology and keyword sorting number labeling.

**Object**	**Person**						**Environment**		
**Keyword**	**Duet-dance**	**Slide**	**Whirl**	**Jump**	**Fast**	**Hold**	**Ice field**	**Audience**	**Light**
Number	1	2	3	4	5	6	7	8	9
C-index	4	3.32	2.86	2.71	2.15	1.88	1.27	1.03	0.54

After the labels of the sorting numbers are assigned, each keyword is tested once. The test is based on the C-index in Table 1. The purpose of the detection is to determine whether the labeling of the sorting number is accurate. Otherwise, the sorting number labeling of the main body and the sub-body will be reassigned. If multiple sub-ontologies and more keywords are determined from the demand information, the ranking numbers are marked sequentially. Table 1 is used to find dance clips from the action library.

**Figure 4:**
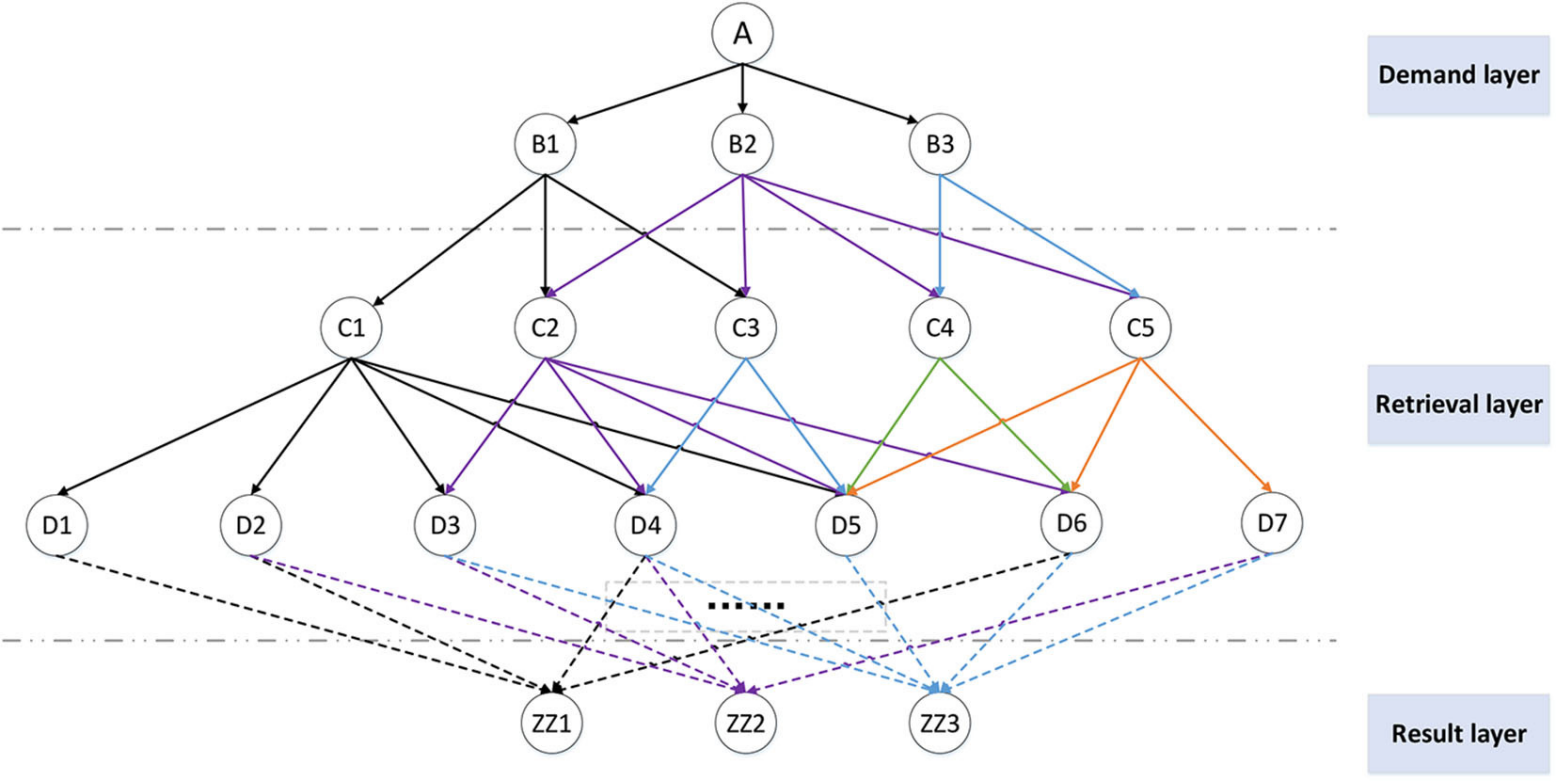
Hierarchical structure of the M-3DQKG model.

The generation of the action library relies on the R-CNN network to extract and label the features of videos, pictures, and audios. The tags are constructed into a knowledge network, as shown in Figure 4. In the network, the demand layer is the ontology information in Table 1. The retrieval layer is the knowledge network in the action library. The resulting layer is the conclusion data generated by knowledge reorganization. The system sequentially calculates the connection weight of the upper level knowledge and the lower level knowledge in the knowledge network. Take the hierarchical structure and node distribution in Figure 4 as an example.

First, determine the interpretation matrix *A* of the primary ontology. According to the searching results of the row labels constructs the matrix, where *B*_1_, *B*_2_, and *B*_3_ connected to it and *B*_2_ > *B*_3_ > *B*_1_. The subordinate ranking numbers are marked as *B*_1_ = 5, *B*_2_ = 2, *B*_3_ = 3.

(1) $$\begin{array}{l} \text{A}=\left[\begin{array}{c} 1\;|1/\left({\text{B}}_{1}-{\text{B}}_{2}\right)||1/\left({\text{B}}_{1}-{\text{B}}_{3}\right)|\\ \left|{\text{B}}_{1}-{\text{B}}_{2}|\;1\;|{\text{B}}_{3}-{\text{B}}_{2}\right|\\ |{{\text{B}}_{1}-\text{B}}_{3}|\;|1/\left({\text{B}}_{3}-{\text{B}}_{2}\right)|\;1 \end{array}\right]\;\overset{\Leftrightarrow }{\;}\text{A}=\left[\begin{array}{c} 1\;1/3\;1/2\\ 3\;1\;1\\ 2\;1\;1 \end{array}\right] \end{array}$$

Calculate the primary weight *W* by the square root method,

(2) $$\begin{array}{l} \text{W}=\left[\begin{array}{c} \sqrt[{3}]{1^{*}|\frac{1}{\left({\text{B}}_{1}-{\text{B}}_{2}\right)}{|}^{*}|\frac{1}{\left({\text{B}}_{1}-{\text{B}}_{3}\right)}|}\\ \sqrt[{3}]{\left|{\text{B}}_{1}-{\text{B}}_{2}{|}^{*}1^{*}|{\text{B}}_{3}-{\text{B}}_{2}\right|}\\ \sqrt[{3}]{|{{\text{B}}_{1}-\text{B}}_{3}{|}^{*}|\frac{1}{\left({\text{B}}_{3}-{\text{B}}_{2}\right)}{|}^{*}1} \end{array}\right]=\left[\begin{array}{c} 0.338\\ 1.442\\ 1.260 \end{array}\right] \end{array}$$

**Figure 5:**
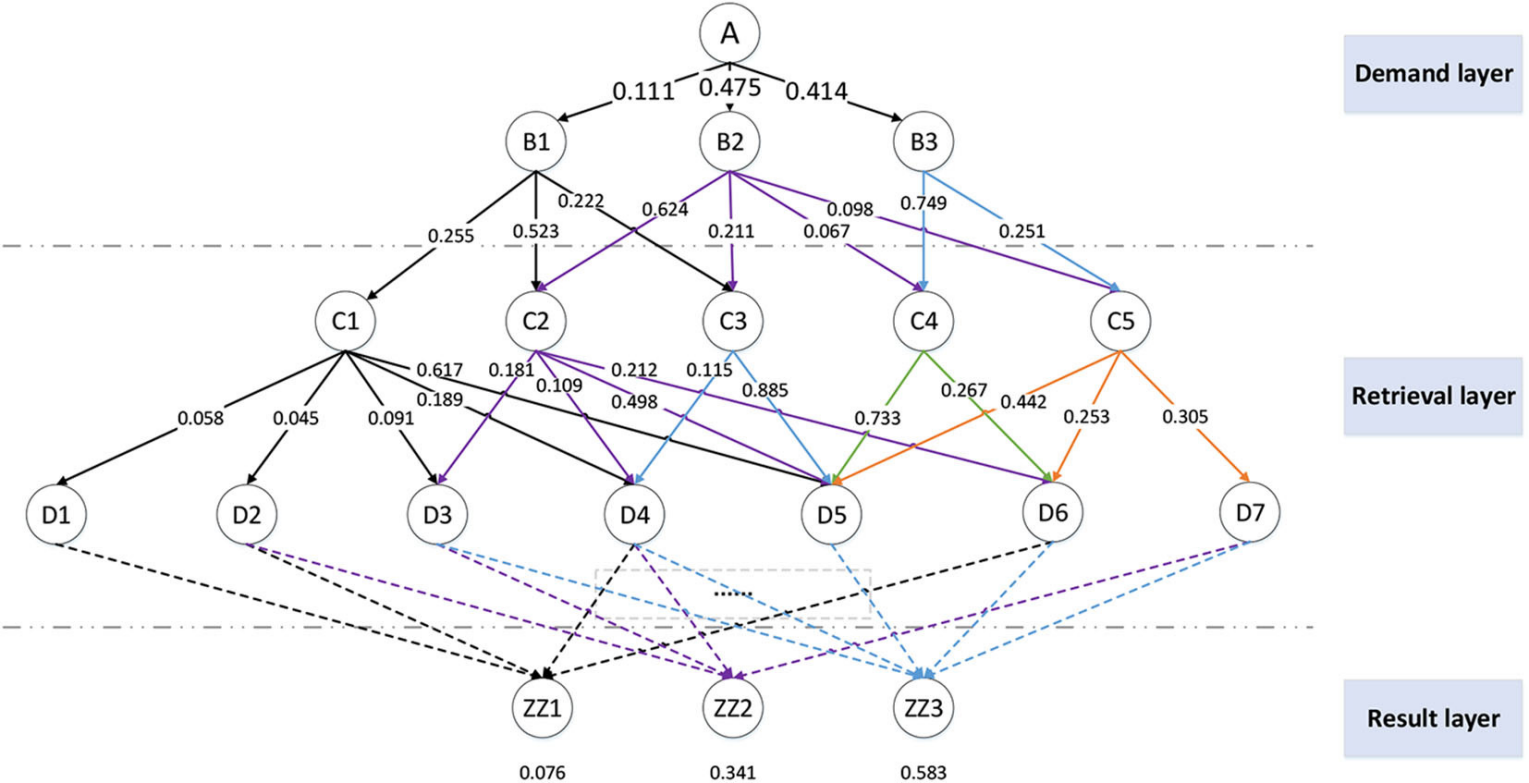
M-3DQKG model retrieval weight calculation and creative library generation.

Normalize *W* using dispersion normalization and map the value between [0, 1],

(3) $$\begin{array}{l} {\text{w}}_{\text{i}}^{*}=\frac{{\text{w}}_{\text{i}}}{\sum_{\text{i}=1}^{\text{N}}{\text{w}}_{\text{i}}} \end{array}$$

(4) $$W=\left[\begin{array}{c} w_{1}^{*}\\ w_{2}^{*}\\ w_{3}^{*} \end{array}\right]=\left[\begin{array}{c} 0.111\\ 0.475\\ 0.414 \end{array}\right]$$

where $w_{i}^{*}$ is the normalized value of each weight and *W*° is the normalized matrix. We can use this method to obtain the retrieval weight between each layer of tags. Mark the weights of each layer on the graph, as shown in Figure 5. It can be seen that, after knowledge retrieval and reorganization, the result layer data are output, and the weight that the knowledge unit can adopt is marked. The weight value can reflect the admissibility of the knowledge unit. The dotted line in the figure indicates that the intermediate knowledge retrieval level is omitted.

**Figure 6:**
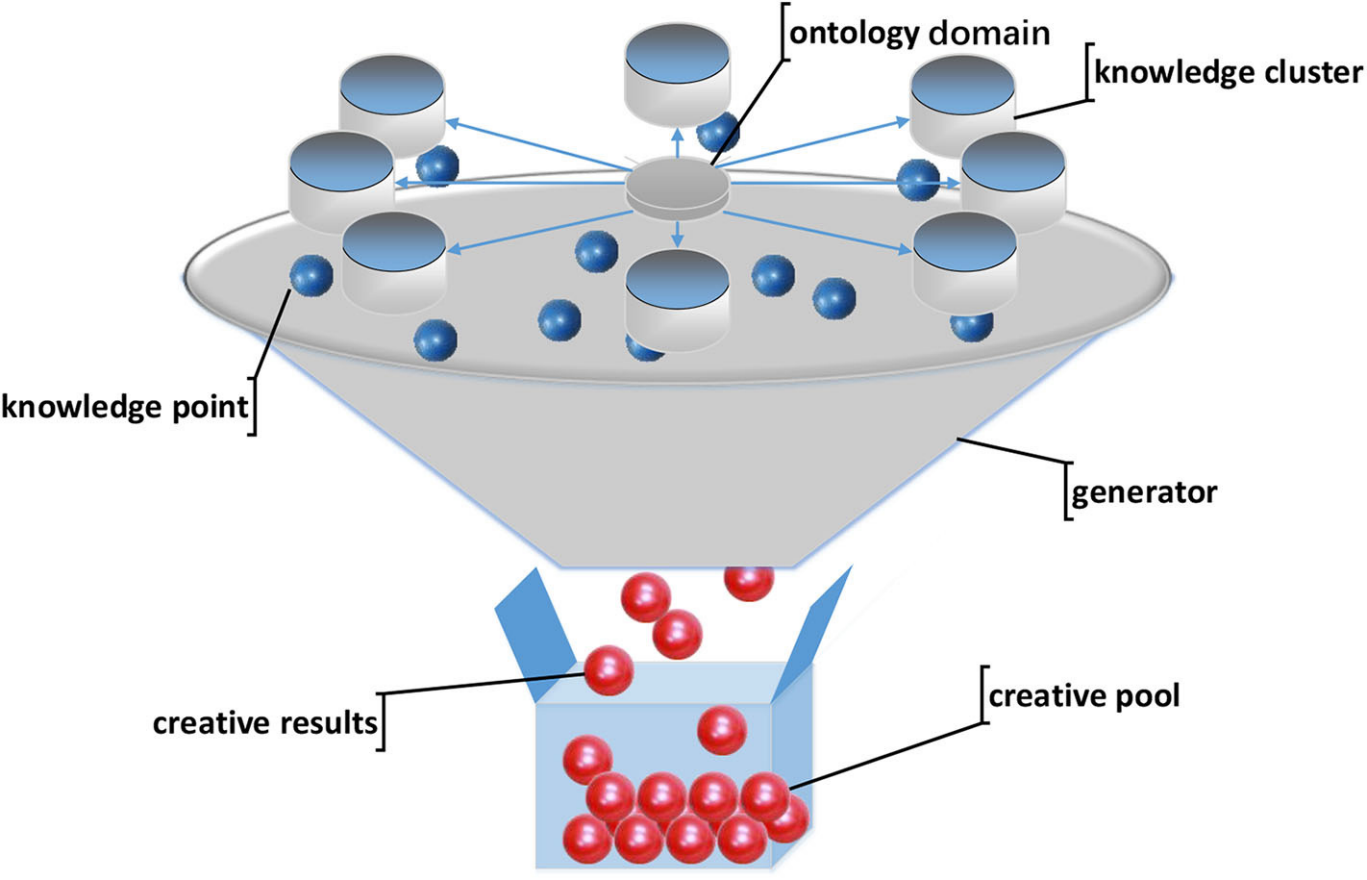
The structure of the probability branch model in the knowledge base.

The knowledge units obtained in the result layer are used to generate a creative library through clustering. The generation of the creative scheme adopts the “probability branch model” to generate the knowledge units in the creative library through combination, as shown in Figure 6. The final scheme judges its validity according to the value of the sum of weights. The M-3DQKG model is repeatedly reconstructed through the continuous “learning–improvement–re-learning–re-improvement” process of the learning network to obtain the most reasonable knowledge unit storage and retrieval structure.

The “probabilistic branching model” in Figure 6 can reorganize the knowledge units of the result layer according to probability. The creative scheme generated after the reorganization is a random combination of “hot knowledge” with a relatively large weight and “cold knowledge” with minimal weight. The model is based on the creative knowledge unit from reorganization at the two levels of quantity and quality, so it improves the novelty and diversity of creative generation schemes.

### Information Fidelity and Creative Generation Model

This paper proposes the concept of “information fidelity” in the process of creative generation. “Information fidelity” means that the information in the system should be as authentic and complete as possible. The information fidelity in this paper includes the reliability of the demand ontology information and the integrity of the information in the information-processing procedure. The former ensures that the results of the creative generation meet the constraints of the ontology domain, while the latter can maximize the creative ability of the system through rich information.

The input system of the required information in the creative generation process is the starting point; it ends with the generation of the creative plan. The process always follows the principle of information fidelity, and, under the premise of satisfying the ontology domain, it emphasizes that creativity should follow natural laws and social reality. Its purpose is to enable creative results to be used to solve a social problem.

Information fidelity needs to reduce the process of extraction and visualization in the process of information processing and transmission. Moreover, based on the current information-processing environment, even if we use multiple threads to process information, we cannot adequately meet the impact of big data. Information will be lost in a specific link, causing information loss. The advantage of parallel quantum computing lies in solving these problems. Creative computing has been able to satisfy the composition of music (Hugill, 2014; Giraud et al., 2016) in music creation, but it is a new endeavor in scene generation and dance performance. Because of the numerous elements involved in the creative calculation and the enormous amount of calculation required, the calculation cannot solve all creative problems in a short time, and the process of composing music is relatively simple.

To ensure the fidelity of the information, we have carried out a lot of research work mainly on the credibility of the information source, the closure of the information-processing method, the integrity of the information-processing results, and the timeliness of the information. Define the information fidelity *I*(*x*) as

(5) $$\begin{array}{l} I\left(x\right)=\;f\left(Sc,\;Cl,Inte,Tim\right) \end{array}$$

where *Sc* is the credibility of the information source, *Cl* is the degree of closure of the information-processing method, *Inte* is the completeness of the information result, and *Tim* is the timeliness evaluation value of the information.

(6) $$\begin{array}{l} Sc\left(x\right)=q\left(1-p\right)\sqrt{\overset{q}{\underset{i=1}{\sum }}\frac{{\left(x_{i}-x_{0}\right)}^{2}}{q}} \end{array}$$

where *p* represents the error tolerance rate of the dataset and *q* is the amount of the subset of the dataset. Sources of information include the initial graphics, audio–video datasets, databases, and requirements documents. All of these must form an exact information index database, which is convenient for checking that the information is correct.

(7) $$\begin{array}{l} Cl\left(m,n\right)=dec\left(m,n\right)\frac{{deep}^{*}num}{sum}{}^{*}{rate}^{*}100\% \end{array}$$

where *dec*(*m, n*) is the information loss function affected by the environment, *m* is the amount of quantum parallel computation, *n* is the number of the structural unit of each parallel computing process, *deep* represents the depth of knowledge graph retrieval, *num* is the cumulative number of knowledge nodes that the process goes through, and *sum* is the total number of nodes of knowledge points. Furthermore, *rate* is the coverage rate of knowledge points. During information processing, the database must not only cover enough demand information but also form a complete expression of the characteristics of the data. Based on the M-3DQKG model construction method in the previous section, the system assigns weights to the knowledge units containing the information of the ontology and realizes information retrieval.

(8) $$\begin{array}{l} Inte\left(t\right)=\frac{\;sets\left(t\right)\cap \left(dm\cup F\right)}{dm\cup F}{}^{*}com{\left(t\right)}^{*}100\% \end{array}$$

where *sets*(*t*) is the fitting function of the dataset containing the ontology domain and feature labels returned at a random time, *dm* is the ontology domain set, *F* is the feature label set, and *com*() is the proper function of the knowledge nodes used. This paper proposes a comparison method to compensate for information loss. The method is a combination of information feedback and a suitable comparison. The feedback information is the ontology domain and feature label of the current creative generation at a random time in the process of generating results. Based on the feedback from these two characteristics, the deep learning network compares them with the original demand data. If the ontology domain or the feature labels are different during the fitting process, it is considered that a suitable comparison has failed. At this time, the system considers that the result of the creative calculation has deviated. This deviation is part of the unsuccessful suitable comparison segment. The successful part of fitting and matching forms the starting value of the creative evaluation score (between [0, 100]). This value will also be used as the evaluation value for the fidelity of the information when the creative generation result is evaluated. Also, all information in the creative process should be kept up to date.

(9) $$\begin{array}{l} Tim\left(t\right)=\frac{\Delta q}{\Delta Q}\frac{\left(t-t^{\prime }\right)}{t}\int_{t}^{t^{\prime }}f\left(x\right)dx \end{array}$$

where *t*−*t*′ is the difference between the current time and the data update time, *q* is the sum of the absolute value of the increase and decrease of the data from *t*′ to *t*, *Q* is the total amount of current information, and *f*(*x*) is the quantization function of the information.

**Figure 7:**
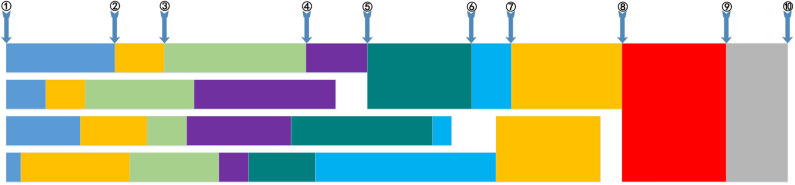
Information-processing timelines.

In the process of generating ideas, the data are updated continuously. The arrows in Figure 7 indicate the input of new data, and the numbers indicate the order of information input. Each row represents the progress of the data calculation. Different colors indicate the module unit of varying processing times. The first line is the primary process, which is used to interact with the data and integrate the data directly. The module unit processes integrated data based on the last completed process. Processes completed before this base point choose to wait. Moreover, the integrated data are used for idea generation.

Creative generation is based on information fidelity calculations. This paper proposes a basic framework for the creative generation model based on the flow of information. The creative generation model was mentioned at the outset of digital creative technology (Lee, 2016), but, unfortunately, the definition of the creative model is relatively one-sided and not universal. Moreover, the method is single and cannot complete the creative generation process quickly and intelligently. This paper uses a variety of deep learning networks and quantum technologies to reconstruct the creative generation model. Compared with previous studies, the framework has the characteristics of universality, high performance, and intelligence.

**Figure 8:**
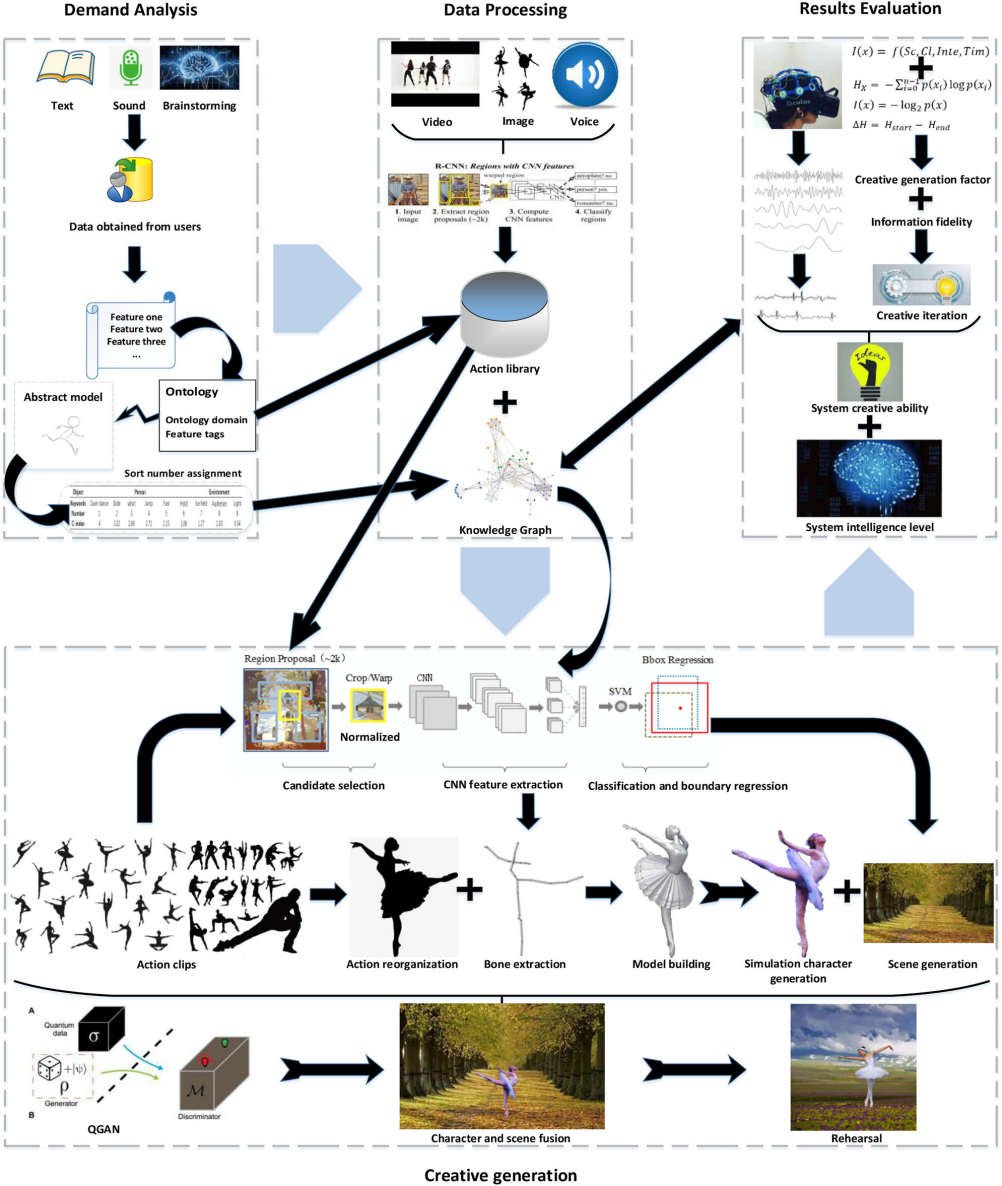
The creative generation model of dance movements integrated with performance scenes. CNN, convolutional neural network; SVM, support vector machines; QGAN, quantum generative adversarial network.

It can be seen from Figure 8 that this paper proposes a four-stage simulation framework for the creative generation process. The demand analysis module determines the ontology domain and feature labels through demand analysis. The system generates abstract models with unified characteristics and stores knowledge weight tables. The data-processing module extracts the action library and knowledge network through the R-CNN network and establishes a connection between them. The M-3DQKG structure is constructed using quantum modeling and simulation methods to realize entangled interconnection and the teleportation of information. The information stored in the qubit forms a backtracking table, which compares the requirements with the update of the knowledge network. In the creative generation module, action learning extracts dance action models through R-CNN and HED and builds simulation models in a 3D engine. At the same time, QGAN generates an expression environment that meets the characteristics of the dance and realizes the integration of dance movements and the expression environment. The mature performance data are twinned on the robot to drive the robot's dance expression. The result evaluation module uses EEG tests to mark the creative tags that trigger excitement on the creative generation results and uses information fidelity and CGC to evaluate the novelty, practicality, and intelligence of the creative results. Under the framework of this creative generation model, computer simulation technology reduces the event cost of robot debugging. It ensures the closure of the creative generation process and the practical value of the creative results.

### Linear Evaluation of System Creative Ability

Three crucial indicators comprehensively evaluate the creative ability of the system. The first one is the amount of information that the system may need to generate originality. The second one is the timeliness of the results, and the last one is the diversity of results. The M-3DQKG model solves the problem of result diversity. Information fidelity is used to evaluate the timeliness of the generated results. Moreover, we study the problem of the calculation of the amount of information in this section. The evaluation of creative results should conform to four criteria. First, the semantic extracted features and demands need to match with others to confirm that the learning network in the creative generation model satisfies the characteristic value of the ontology domain. The M-3DQKG model does this work. Second, the abstract model and dataset training results also need to match to generate an abstract model based on the dataset that has the same attributes as the research object. The creative generation model solves this problem. Third, the output results and realization conditions need to match to ensure that creative solutions can be realized under existing conditions. Information fidelity computing achieves this goal. Finally, the creative ability and psychological expectations need to match to develop a creative generation system that meets people's expectations. This issue will be studied in this section.

**Figure 9:**
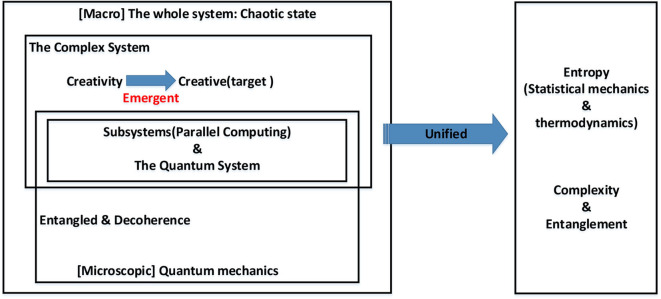
The relationship between macroscopic complex systems and microscopic quantum systems.

This paper proposes a method to evaluate the creative ability of the system by calculating the complexity of the system to quantify the creative capacity of the system. This method maps the non-linear relationship of the complex macroscopic system into the linear space of the microscopic quantum system. The complexity of the system is quantitatively analyzed by calculating the changes in system energy during information transmission and conversion. The amount of information calculates the measurement of information. Entropy is used as a unified calculation standard to quantify the energy change in the system from the thermodynamics perspective (Nalewajski, 2016). Thereby, humans can obtain system complexity (Burdon et al., 2018). As shown in Figure 9, the entropy is used to measure the consistency between macro-complex systems and micro-quantum systems. At the same time, this paper proposes the concept of the “creative generation coefficient” (CGC), which is a standardized value used to map the system's current complexity and information volume. The value continually changes with time. What is more, the creativity generation coefficient is just like the value of evaluating people's IQs. It can also reflect the level of intelligence and creativity of the system. When the CGC reaches a specific value, it means that the complexity of the system has reached a high level. According to thermodynamic theory, the increasing trend is suppressed by quantum effects, although quantum chaos can lead to an increase in entanglement, that is, an increase in quantum entanglement entropy. In other words, the entanglement entropy cannot be infinite. It tends to converge. Humans cannot make the complex system lose its original emergent properties by increasing the entanglement of the quantum system. The entropy of the system cannot be increased indefinitely, which also coincides with the emergence of complex systems (Morrison, 2003). If the system emerges strongly, it gradually changes from the disordered state to the ordered state when the emergence process occurs. Then, the entropy of the system decreases. Therefore, we define that the system will emerge and complete the creative generation process when the CGC grows to a higher value.

Information is stored in qubits, which are particles. They are in a linear superposition state. This can be expressed as

(10) $$\begin{array}{l} \Psi ={\text{c}}_{1}\Psi_{1}+{\text{c}}_{2}\Psi_{2} \end{array}$$

where **Ψ**_1_ and **Ψ**_2_ are the possible states of particles and *c*_1_ and *c*_2_ are constant complex numbers. If entanglement occurs in the quantum system, the possible state of the particles can be expressed as a spin form, which is shown with the Dirac mark

(11) $$\begin{array}{l} \left|\;\psi \rangle \;=\;\frac{1}{\sqrt{2}}(|\;↑\rangle \;⊗|\;↓\rangle \;-|\;↓\rangle \;⊗|\;↑\rangle \;\right) \end{array}$$

where | ↑〉 and | ↓〉 indicate that the spin of the particles is up or down, respectively. In order to facilitate calculation, quantum entanglement is expressed mathematically. Assuming that a composite system is composed of two subsystems A and B, the Hilbert spaces of these two subsystems are *H*_*A*_ and *H*_*B*_, respectively, and the Hilbert space *H*_*AB*_ of the composite system is a tensor product

(12) $$\begin{array}{l} {\text{H}}_{\text{A}\text{B}}={\text{H}}_{\text{A}}⊗{\text{H}}_{\text{B}} \end{array}$$

Set the quantum states of subsystems A and B to | α〉_*A*_, | β〉_*B*_, respectively. If the quantum state of the composite system | ψ〉_*AB*_ cannot be written as a tensor product | α〉_*A*_ ⊗ | β〉_*B*_, the compound system is called the entanglement system of subsystems A and B, and the two subsystems A and B are entangled with each other.

The state vector | ψ〉 or the density operator ρ = | ψ〉 〈 ψ| can describe the quantum state of a pure state. Density operators can only describe statistical mixed states

(13) $$\begin{array}{l} \rho =\underset{\text{k}}{\sum }{\text{P}}_{\text{k}}|\;\psi_{\text{k}}\rangle \;\langle \;\psi_{\text{k}}|\; \end{array}$$

where *P*_*k*_ is the pure state | ψ_*k*_〉 probability of occurrence in the statistical mixed state ρ. They satisfy the normalization condition

(14) $$\begin{array}{l} \text{Tr}\rho =\underset{\text{k}}{\sum }{\text{P}}_{\text{k}}=1 \end{array}$$

Because of the inevitable effect of the environment, the system is converted from the initial pure state (the coherent superposition state of the coherent state) to the mixed state (the incoherent superimposed state of the coherent state) when passing through the cavity field in the process of information propagating using the qubit as a carrier. The pure state of this composite system can be expressed as

(15) $$\begin{array}{l} \left|\;\psi \left(0\right)\rangle \;\propto (|\;\alpha \rangle \;+|\;-\alpha \rangle \;\right) \end{array}$$

where | α〉 is an assumption that the cavity field is initially prepared in a coherent state and defines that the environment is initially in the state | ε〉, then the state of the composite system at time *t* becomes

(16) $$\begin{array}{l} \left|\;\psi \left(\text{t}\right)\rangle \;\propto (|\;\beta \left(\text{t}\right)\rangle |\;\varepsilon_{1}\rangle \;+|\;-\beta \left(\text{t}\right)\rangle |\;\varepsilon_{2}\rangle \;\right) \end{array}$$

where

(17) $$\begin{array}{l} \beta \left(\text{t}\right)=\alpha {\text{e}}^{-\frac{\gamma \text{t}}{2}} \end{array}$$

γ represents the information loss rate of the cavity field and | ψ〉 is the quantum state of the cavity field. To trace the environmental variables, the reduced density operator of the cavity field is

(18) $$\begin{array}{l} \rho_{\text{F}}={\text{T}\text{r}}_{\text{e}}\rho =\underset{\text{j}}{\sum }\langle \varepsilon_{\text{j}}|\psi \left(\text{t}\right)\rangle \langle \psi \left(\text{t}\right)|\varepsilon_{\text{j}}\rangle \propto |\;\beta \left(\text{t}\right)\rangle \;\\ \;\langle \;\beta \left(\text{t}\right)|\;+|-\;\beta \left(\text{t}\right)\rangle \;\langle -\;\beta \left(\text{t}\right)| \end{array}$$

It can be seen that, because of the effect of the environment, the pure state becomes a mixed state. When *t* → ∞, the cavity field information will decay to the vacuum state |0〉. For linear entropy, the nature of the trace of the density operator ρ is

(19) $$\begin{array}{l} \text{Tr}\rho =\{\begin{array}{c} 1,\;\text{pure state}\\ <1,\;\text{mixed state} \end{array} \end{array}$$

Tracing the density operator can obtain the moisture entropy of the system, then the entropy value of the system is calculated according to the entanglement entropy

(20) $$\begin{array}{l} \text{S}\left(\rho \right)=-\text{T}\text{r}\left(\rho \log\rho \right) \end{array}$$

The rate of change of entropy with time is

(21) $$\begin{array}{l} \dot{\text{S}}=-\frac{\text{dTr}\rho^{2}}{\text{d}\text{t}}=-\text{T}\text{r}\left(\dot{\rho }\rho +\rho \dot{\rho }\right) \end{array}$$

Since the entanglement entropy is not additive, the amount of information reflecting the two entangled subsystems is

(22) $$\begin{array}{l} \text{I}\left(\text{A},\text{B}\right)=\text{S}\left(\text{A}\cup \text{B}\right)-\text{S}\left(\text{A}\right)-\text{S}\left(\text{B}\right)+\text{S}\left(\text{A}\cap \text{B}\right) \end{array}$$

This is Shannon's proposed definition of information entropy. It is seen that the information entropy can be expressed as the mathematical expectation of the amount of information *I*(*x*_*i*_) provided in the system when an event *x*_*i*_ appears in set *X*, which is

(23) $$\begin{array}{l} {\text{H}}_{\text{X}}=\;-\overset{\text{n}-1}{\underset{\text{i}=0}{\sum }}\text{p}\left({\text{x}}_{\text{i}}\right)\log\text{p}\left({\text{x}}_{\text{i}}\right) \end{array}$$

For information, it is a question of probability. A large probability only requires one bit to be transmitted. In other words, the smaller the probability, the more bits need to be transmitted. Using the number of bits to measure the amount of information, the lower probability, the enormous amount of information. The formula based on this information is

(24) $$\begin{array}{l} I\left(x\right)=-log_{2}p\left(x\right) \end{array}$$

It can be clearly seen that there is a correlation between entanglement entropy and information entropy. The change process of the system from the pure state to the mixed state also exactly meets the necessary environment for creative computing (Liu and Yang, 2014). The amount of information on the two entangled subsystems can also be calculated. When the input information is stored in the qubit unit of the system, the amount of information is known. Using this as the cardinal number, humans can change the system information through the calculation used in the process of information transmission. This change is mainly the result of the increasing amount of information. The added information is the process data and this results in the dataset generated by the learning network after learning. It also includes a knowledge graph that represents the relationship between the data, an abstract model representing the ontology of things, and point information, representing the movement of the model. When the amount of information reaches a specific value, the information entropy of the system also reaches a higher value. At this time, as a subsystem of a complex system, each quantum system can map the result to the information entropy of the complex system through the calculation of information entropy. Based on this, the complexity of a complex system can be measured. For this measure of complexity, the value can be mapped to a value between [0, 100]. This is the “creative generation factor” (CGC) that we proposed. The emergence phenomenon will occur when the system information is saturated. At this time, the system information is continuously released to the outside system, and the information entropy of the system will increase slowly, and even tend to converge. The quantity of emergence of the system is defined as the total value from the beginning of emergence to the end of it, that is,

(25) $$\begin{array}{l} \Delta H=\;H_{start}-H_{end} \end{array}$$

This can more intuitively represent the system's ability to generate creativity. The CGC *C*(*x, t*) is

(26) $$\begin{array}{l} \text{C}\left(\text{x},\text{t}\right)\to \text{f}\left(\text{I},\;\Delta \text{H},\;{\text{H}}_{\text{X}},\dot{\text{S}}\right) \end{array}$$

When the system emerges, the CGC current value is called the “emergence value range” (EVR). After the CGC reaches the EVR, the representation function of the CGC tends to converge. From the perspective of system evaluation, we believe that the system has a high creative generation ability. We use this method to achieve a linear evaluation of the system's ability to generate originality. This method, combined with EEG, can reasonably evaluate the results of the creative generation. We will explain the evaluation experiment process in detail in the next section.

## Results

The experiment was deployed on an IBMAC922 machine, using four Tesla V100 floating-point arithmetic cards. It was compiled with Python3.5+ and OpenCV 3.4 under the environment of IBMpcc64 ubuntu18.04. The experiment used the cityscape database for 1,000 epochs of deep learning. It was able to obtain 3D scenes and task models, generate action libraries, and perform fragmented creative simulations of dance movements. The engine achieves dance performances by combining creative dance moves and generated simulation scenes. Then, the system gets the relevant results, showing that the generated actions are not the same as the learned video library. The rearrangement of the combined sequence of actions and the movement path of the skeleton of the simulation model can achieve creative generation. Dance movements can be viewed through VR devices, and humans can experience the viewing effects in the performance scene.

According to different task requirements and files, the learning network extracts data feature values and labels the data to form the original feature label table, as shown in Table 2. Dance creativity is classified according to the type of dance, such as folk dance, tap dance, and ballet. We assign keyword tags that represent the characteristics of each type of dance. These tags are used to match with the requirements to determine the actions needed for that dance action. Moreover, dance performances combined with dance moves need to have corresponding scenes to express the artistic conception. Therefore, the environmental label is also stored in this table. For example, mountains, plants, landscapes, and street scenes. The data extracted from this table are as accurate as possible. As the only constant table, it is the standard for feedback comparison in the system learning network's learning process, and it is also the basis for data review. Similarly, this table contains not only the primary data for defining the ontology domain of ideas but also provides the basis for evaluating the results of creative generation.

**Table 2:** Original feature labels.

**Ontology**	**Feature label**	**Attributes**	**Layer number**	**Node number**	**Retrieval rate**
Folk dance	Dai people	Peacock, drums, performance…	6	541	0.673
	Dong nationality	Chorus, lusheng, love…	6	532	
	Korean	Sword dance, fan dance, chic, cheerful…	5	344	
	Uighur	Plate dance, tambourine dance, head, shoulders, waist, and eyes…	8	892	
	…	…			
Tap dance	American	Free, relaxed, complex rhythm, jazz…	3	87	0.522
	British	Rotate, slide, beautiful, split sound, repeat…	5	381	
	Irish	No upper body movements, crossed feet, soft shoes…	3	98	
	…	…			
Ballet	Banquet ballet	Song, dance, recitation…	2	22	0.735
	Court ballet	Drama, acrobatics, music…	2	19	
	Plot ballet	Education, story…	4	235	
	Romantic ballet	Lighting, big screen, lightness, poetry…	3	106	
	Russian ballet	Women, elves, ghosts, gods, longing…	4	269	
	Contemporary ballet	Life, stretch, jump, straight…	3	112	
	…	…			
**…**					
Mountains	Green hills	Green, vitality, birds, and flowers…	5	401	0.449
	Snow mountain	White, cold, steep…	3	115	
	Distant mountain	Small, rolling…	5	384	
	……	…			
Plants	Flower	Colorful, petals, stamens, chrysanthemums, sunflowers…	4	298	0.907
	Grass	Green, four-leaf clover, mimosa…	7	754	
	Tree	Colors, leaves, branches, trunks, pine trees, willows…	5	372	
	…	…			
Landscapes	Bright moon	Bright, curved, round…	2	13	0.846
	Mountains	High, overlapping…	6	557	
	Morning glow	Red, big, halo…	5	369	
	…	…			
Street scenes	Street	Width, length, car, lights…	8	933	0.633
	Building	Shape, floor…	11	1,421	
	Shops	Signboard, display…	10	1,275	
	…	…			
**…**					

For the creative process of dance movements, an initial knowledge graph is formed, according to Table 2. This knowledge graph is the accumulation of experience in the formation of traditional ideas. The system must search and form a new knowledge graph–M-3DQKG search structure, first based on the task feature label. Then, the system stores the knowledge in the qubit. For each knowledge point, the weight is determined according to the degree of primary and secondary requirements, as shown in Table 3. M-3DQKG retrieves the hierarchical management of the structure and defines the weight of the parent node to the lower child node. The sum of the weights is 1. There may be entangled nodes each time, defining the entangled pairs for cross-level retrievals, such as B2 and D9.

**Table 3:** Original table of the knowledge graph and weights.

	**Label**		**Connection nodes**						**Entangled nodes**
**Name**	A	A1	B3	B7	B10				
W			0.241	0.533	0.226				
Name		A2	B2	B5	B9	B12			
W			0.321	0.552	0.109	0.018			
Name		A3	B1	B3	B4	B6	B8	B11	
W			0.116	0.284	0.485	0.061	0.033	0.021	
Name	B	B1	C5	C8	C13	C17			
W			0.702	0.135	0.098	0.065			
Name		B2	C1	C6	C7	C16	C22		D9
W			0.238	0.299	0.374	0.018	0.071		
Name		B3	C3	C4	C11	C15	C16		
W			0.096	0.167	0.355	0.219	0.163		
Name		B4	C6	C8	C12	C15	C20		
W			0.457	0.166	0.182	0.064	0.131		
Name		B5	C4	C9	C14	C18	C24	C31	
W			0.205	0.611	0.034	0.142	0.005	0.003	
…		…							
Name		B11	C2	C7	C19				F11
W			0.857	0.032	0.111				
Name		B12	C5	C11	C27	C32			
W			0.629	0.174	0.062	0.135			
Name	C	C1	D3	D12	D44	D47			
W			0.525	0.261	0.103	0.111			
Name		C2	D2	D23	D39				
W			0.732	0.233	0.035				
Name		C3	D9	D11	D52	D69	D73		E7
W			0.494	0.268	0.127	0.042	0.069		
…		…							

**Figure 10:**
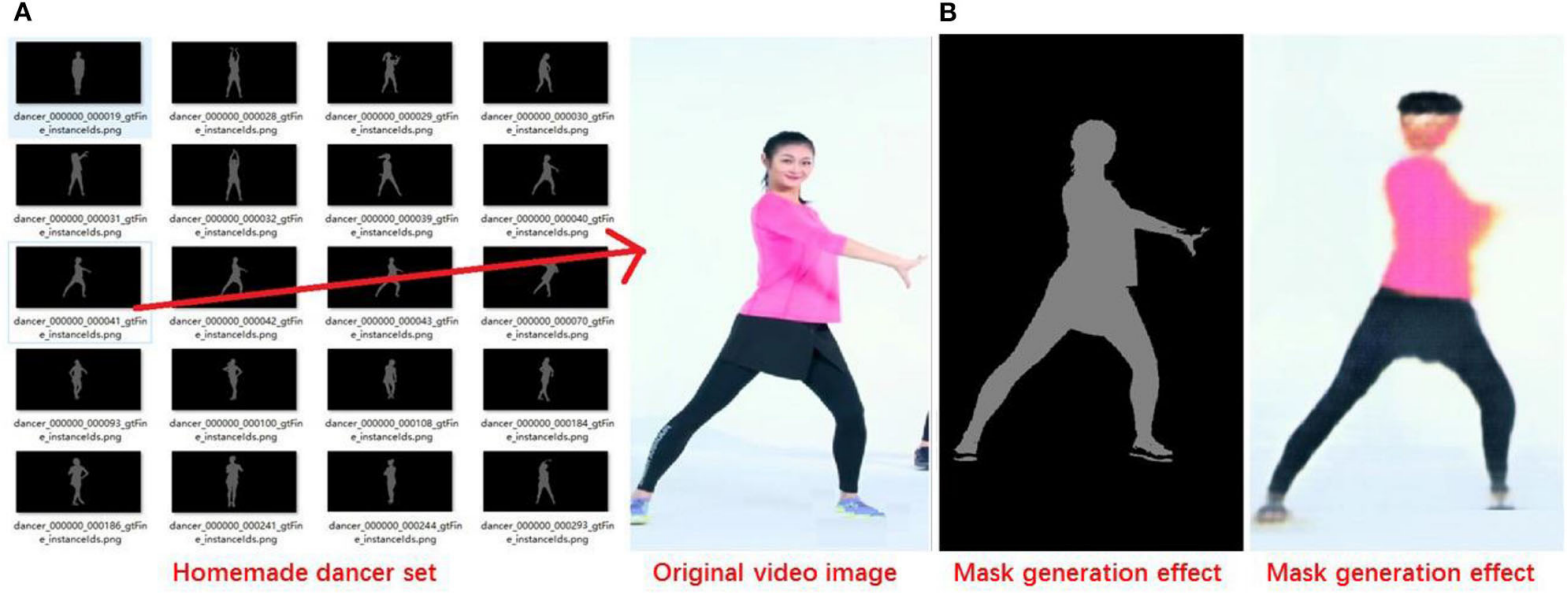
Dance movement generation **(A)**. Deep learning process **(B)**. Generation of virtual people.

According to the weight distribution of the knowledge graph, dance movements are recombined from the action library formed by the learning network through learning to form dance fragments, which are combined with the probabilistic branch model. The system generates simulation scenes based on dance clips and demand ontology domains. Then, the simulation scene and dance movements are combined to complete the creative process. Figure 10 shows an example of generating a character abstract model and the dance movements.

**Figure 11:**
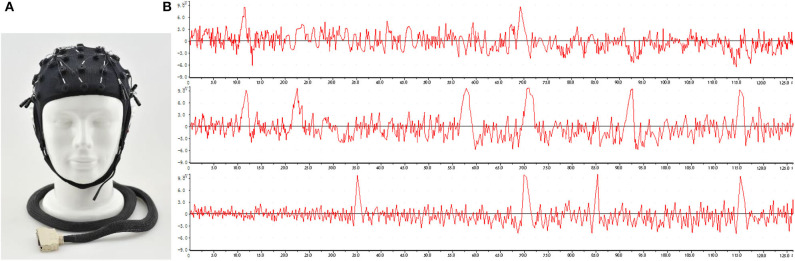
Electroencephalographic (EEG) experiment and waveform. **(A)** Schematic of the EEG equipment used. **(B)** EEG waveforms of three subjects.

The subjects watched the creative generation plan and recorded EEG data through EEG equipment, as shown in Figure 11. The participants were creative needs proposers and student volunteers (a total of 26 people). The former assessed how well the creativity needs are met, while the latter pretended to be an audience watching the creativity. When the subject was interested in the creative points generated by the system, the EEG data obtained from the test had a peak. The time corresponding to the peak was defined as the creative time point. In Figure 11, for example, the creative time points for the first subject were the 12th and 69th s. The subjects watched the creative plan again. This time, they compared their EEG data and marked the creative tag at the point of the stimulus (the peak point during the EEG). We analyzed these times in the subjects' EEG data and marked keywords that were of interest to the subjects. We organized these keywords to form an evaluation form, as shown in Table 4. We added the tags for each subject to the knowledge graph one by one as the negative feedback knowledge of M-3DQKG. If the label already existed in the knowledge graph, then a new round of machine learning updated its weight *W*. After repeated iterations, we perfected the structure of M-3DQKG.

**Table 4:** Test takers' creative input label.

**Creative time**	***T* = 12 s**		***T* = 35 s**			***T* = 84 s**	***T* = 92 s**	
**Insertion time**		***T* = 23 s**		***T* = 57 s**	***T* = 69 s**			***T* = 115 s**
Subject 1	Gesture				Spinning			
Subject 2	Cross	Light		Imagination	Passion		Dynamic	Warm
Subject 3			Bounce		Spinning	Expression		Squat-up
Subject 4	Fly	Light	Power			Pleasant		Elegant
Subject 5				Imagination	Spinning		Music	
Subject 6			Power		Spinning	Pleasant		Soothing
Subject 7		Light			Spinning	Expression	Music	Warm
…								
Subject 26				Imagination	Spinning	Expression	Dynamic	Elegant

As shown in Figure 11, different subjects have different points of interest in creativity. For example, the top subject only showed peaks on both sides, while the middle subject showed peaks that appeared very frequently. However, it can be seen that they have coincident peak points. Table 4 reflects the intersection of the creative points.

According to the evaluation results, the previous knowledge graph is readjusted to assign weights to highlight the creative points of the test-takers' focus. The results of one adjustment are shown in Table 5. Compared with Table 3, the connection node of the A1 node is increased by B31, and the connection weight of each node has changed. Similarly, the number of connected child nodes of other parent nodes has been adjusted. As the creative label in Table 4 was added on the one layer, the number of changed nodes increased, and the numbering sequence was changed. Such adjustments may need to be made hundreds or thousands of times. All are done automatically by the system. Figure 11 shows the EEG test results of the first three test-takers in Table 4.

**Table 5:** Change table of the knowledge graph and weights.

	**Label**		**Connection nodes**						**Entangled nodes**
Name	A	A1	B3	B7	B10	B31			
W			0.203	0.515	0.126	0.156			
Name		A2	B2	B5	B12				C6
W			0.333	0.585	0.082				
Name		A3	B1	B3	B4	B6	B8	B11	
W			0.116	0.284	0.485	0.061	0.033	0.021	
Name	B	B1	C5	C8	C14	C19	C31		
W			0.513	0.089	0.114	0.065	0.219		
Name		B2	C1	C6	C11				D21
W			0.388	0.305	0.307				
Name		B3	C3	C4	C11	C15	C16		
W			0.096	0.167	0.355	0.219	0.163		
Name		B4	C12	C23					
W			0.698	0.302					
Name		B5	C4	C9	C14	C18	C24	C31	
W			0.205	0.611	0.034	0.142	0.005	0.003	
…					…				
Name		B11	C2	C7	C19	C39			F16
W			0.646	0.014	0.125	0.215			
Name		B12	C5	C11	C27	C32	C33		D54
W			0.352	0.109	0.262	0.135	0.142		
…					…				
Name		B16	C33	C36					
W			0.732	0.268					
Name	C	C1	D3	D12	D44	D47	D66		G78
W			0.455	0.198	0.117	0.102	0.128		
Name		C2	D2	D23	D52				
W			0.227	0.532	0.241				
Name		C3	D12	D16	D56	D71	D75	D81	E11
W			0.387	0.206	0.158	0.033	0.012	0.204	
…					…				

**Figure 12:**
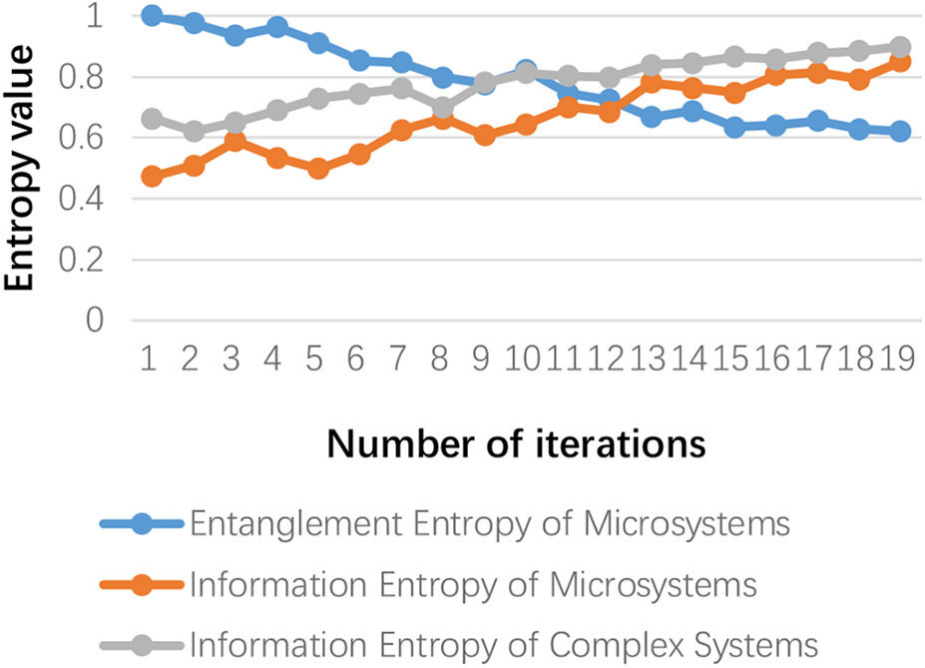
Entanglement entropy, quantum system information entropy, and complex system information entropy.

**Figure 13:**
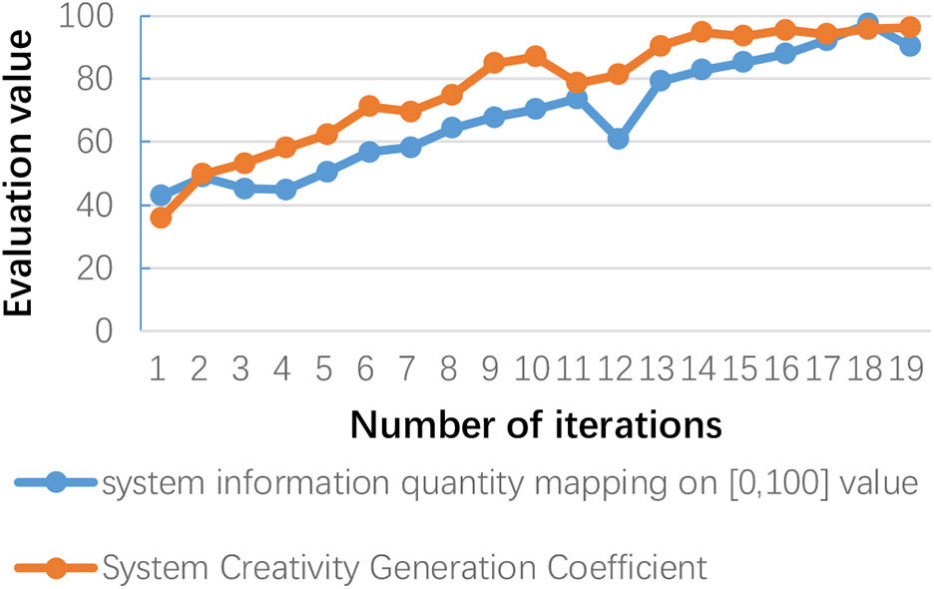
Information volume and creative generation coefficient.

Finally, the creative results that meet the creative needs are evaluated for their information fidelity. Meanwhile, in the creative generation process, we have to evaluate the system's creative generation ability. As shown in Figure 12, the entanglement entropy and information entropy of the microscopic system are tested, and the two are mutual proof that errors in the measurement data have been prevented. Similarly, the information entropy of a complex system is calculated through the change in information volume of the macro-system. It can be seen that there is a correlation between the three. It can be proved that the calculation of the information entropy of the complex macroscopic system can be obtained by the linear calculation of the microscopic quantum system. Based on the calculation method of the CGC proposed in this paper, the information entropy of the complex system is mapped to the interval [0, 100], as shown in Figure 13. It is not difficult to see that there is also a mapping relationship between the CGC, information entropy, and system complexity entropy. Furthermore, it is used as the basis to calculate the creative generation ability of complex systems.

As shown in Figures 12, 13, the mapping relationships among the CGC, the system information quantity, and the system complexity have provided the quantitative calculation basis for the assessment of the system's creative ability. When the CGC reaches an absolute value, the amount of information will no longer increase significantly, and tends to converge. As shown in Figure 13, after 15 iterations per month, the value is stable at around 95. This phenomenon can be explained by the heating effect of the metering subsystem. Currently, the system completes the creative generation process and releases some storage space. In other words, the process of releasing energy in a complex system is the process of completing the emergence of information and realizing the creation of ideas. In this way, the pressure on the system caused by process data storage during the calculation process is relieved. The performance of the system is much improved. It can also be seen that the creative generation ability of the system has been maintained at a high level in the subsequent creative generation process.

The EEG waveform and the subject's creative label annotations reflect the popularity of the current creative system very well. As the tag knowledge is learned again, the structure of M-3DQKG tends to be reasonable. The next creative plan will still be marked by the subject's EEG test results and creative tags as one of the criteria. The loop of this process may require multiple iterations. In the process of system iteration, the information entropy of the system increases continuously and tends to be saturated. Therefore, CGC steadily approaches 100. When the creative generation results are satisfactory to the subjects, the current CGC is fixed as the system's creative emergence value. At this time, the dance creative M-3DQKG structure that addresses these needs is set. When the system faces new dance creation needs, the dance creativity can be simplified to observe whether the CGC value reaches the creative emergence value, and the process of EEG testing and creative labeling is omitted. Therefore, the whole process of dance creativity can be completed more quickly and effectively. Because of the small number of subjects, the coincidence of EEG peaks between different subjects needs to be strengthened. The time points of some creative point tags obtained from the test may be optimized. Furthermore, in order to accelerate the speed and accuracy of creative generation iterations, the subject technology can be expanded, which is one of the important means of achieving this.

## Conclusion and Discussion

This paper builds a creative generation process based on a quantum modeling simulation framework and proposes a method that can generate multiple creative schemes for dance movements. This method can realize the reasonable construction and reorganization of a quantum knowledge graph and assign knowledge retrieval weights according to creative needs dynamically. The method includes the M-3DQKG model, information fidelity method, the creative generation model, and the linear evaluation method of the creative ability. Furthermore, the learning results from the creative network are novel, useful, and diverse based on this method. The practicality of the dancing robot's movements and running trajectories can also be guaranteed. The results of the creative generation can be viewed through a computer simulation engine and VR devices so that people can feel immersed in the experience. For the results of the creative generation process, this paper proposes CGC to judge the creative generation ability of the system. Moreover, the evaluation result data are input to the dancing robot to realize the robot's dancing performance and trajectory calculation in a specific scene. In this paper, the robot data are generated by simulation technology, and the writing and debugging work is completed in the simulation engine. This is helpful in reducing the time cost and hardware loss of the dance robot's multiple runs and debugging, and can map the real robot in the model of the simulation engine through digital twin technology. The benefits of this are to facilitate the analysis and preservation of data and to ensure information fidelity and intelligent control of the dancing robot. At present, in terms of the performance of dance robots, we have found that, in the process of generating creative dance movements, the continuity of movements needs to be optimized by algorithms because the creative movements are fragmented and the dynamic model movements are rigid. The goal of the next stage is to be able to generate a complete set of coherent and smooth dance movements. In terms of the experimental data of the system, because of the small number of subjects, the EEG and creative label data are not sufficient, resulting in a slow system iteration speed, which affects the current generation effect of dance creativity. The next step will be to invest more research in the process of increasing test data and accelerating iteration.

## Data Availability Statement

The original contributions presented in the study are included in the article/supplementary material, further inquiries can be directed to the corresponding author/s.

## Ethics Statement

This study was reviewed and approved by the Medical Ethics Committee of Laizhou People's Hospital, 2020040700001. Written informed consent was obtained from all participants for their participation in this study.

## Author Contributions

PM was responsible for article writing and experimental work. GD directs the article structure and designs experiments. QJ conducts a lot of research work. FZ was the corresponding author and participates in the revision of the methodology. YJ was responsible for data analysis. All authors contributed to the article and approved the submitted version.

## Conflict of Interest

YJ was employed by the company Beijing Wanshide Technology Co. The remaining authors declare that the research was conducted in the absence of any commercial or financial relationships that could be construed as a potential conflict of interest.
